# Rumen microbial degradation of bromoform from red seaweed (*Asparagopsis taxiformis*) and the impact on rumen fermentation and methanogenic archaea

**DOI:** 10.1186/s40104-023-00935-z

**Published:** 2023-11-01

**Authors:** Pedro Romero, Alejandro Belanche, Elisabeth Jiménez, Rafael Hueso, Eva Ramos-Morales, Joan King Salwen, Ermias Kebreab, David R. Yáñez-Ruiz

**Affiliations:** 1 Estación Experimental del Zaidín, Consejo Superior de Investigaciones Científicas (CSIC), Profesor Albareda 1, Granada, 18008 Spain; 2 Department of Animal Production and Food Sciences, University of Zaragoza, Miguel Servet 177, Saragossa, 50013 Spain; 3 Blue Ocean Barns Inc., Redwood City, CA USA; 4 Department of Animal Science, University of California, Davis, CA 95618 USA

**Keywords:** Bromoform metabolism, Dibromomethane metabolism, Methane mitigation, Methanogens, Rumen microbiota, Seaweed

## Abstract

**Background:**

The red macroalgae *Asparagopsis* is an effective methanogenesis inhibitor due to the presence of halogenated methane (CH_4_) analogues, primarily bromoform (CHBr_3_). This study aimed to investigate the degradation process of CHBr_3_ from *A. taxiformis* in the rumen and whether this process is diet-dependent. An in vitro batch culture system was used according to a 2 × 2 factorial design, assessing two *A. taxiformis* inclusion rates [0 (CTL) and 2% DM diet (AT)] and two diets [high-concentrate (HC) and high-forage diet (HF)]. Incubations lasted for 72 h and samples of headspace and fermentation liquid were taken at 0, 0.5, 1, 3, 6, 8, 12, 16, 24, 48 and 72 h to assess the pattern of degradation of CHBr_3_ into dibromomethane (CH_2_Br_2_) and fermentation parameters. Additionally, an in vitro experiment with pure cultures of seven methanogens strains (*Methanobrevibacter smithii*, *Methanobrevibacter ruminantium*, *Methanosphaera stadtmanae*, *Methanosarcina barkeri*, *Methanobrevibacter millerae*, *Methanothermobacter wolfei* and *Methanobacterium mobile*) was conducted to test the effects of increasing concentrations of CHBr_3_ (0.4, 2, 10 and 50 µmol/L).

**Results:**

The addition of AT significantly decreased CH_4_ production (*P* = 0.002) and the acetate:propionate ratio (*P* = 0.003) during a 72-h incubation. The concentrations of CHBr_3_ showed a rapid decrease with nearly 90% degraded within the first 3 h of incubation. On the contrary, CH_2_Br_2_ concentration quickly increased during the first 6 h and then gradually decreased towards the end of the incubation. Neither CHBr_3_ degradation nor CH_2_Br_2_ synthesis were affected by the type of diet used as substrate, suggesting that the fermentation rate is not a driving factor involved in CHBr_3_ degradation. The in vitro culture of methanogens showed a dose-response effect of CHBr_3_ by inhibiting the growth of *M. smithii*, *M. ruminantium*, *M. stadtmanae*, *M. barkeri*, *M. millerae*, *M. wolfei*, and *M. mobile*.

**Conclusions:**

The present work demonstrated that CHBr_3_ from *A. taxiformis* is quickly degraded to CH_2_Br_2_ in the rumen and that the fermentation rate promoted by different diets is not a driving factor involved in CHBr_3_ degradation.

## Background

The fermentation of carbohydrates in the forestomach of ruminants into volatile fatty acids (VFA) produces dihydrogen (H_2_) and carbon dioxide (CO_2_) as fermentation by-products, which are used by methanogenic archaea in the rumen to generate methane (CH_4_) [1]. The release of enteric CH_4_ into the atmosphere represents a negative impact on the environment as its global warming potential is 28–34 times that of CO_2_ over 20-year horizon, but also a loss of 2%–12% of the gross feed energy from the animal [2]. Both the contribution to greenhouse gas emissions and energy loss justify the need for nutritional strategies to reduce enteric CH_4_ emissions without affecting the efficiency of feed utilization and animal health.

Recent investigations have shown that the use of some feed additives or supplements may provide potent emissions reduction [3]. Among the feed additives, the latest studies have demonstrated the inhibitory potential of certain brown and red macroalgae, particularly the genus *Asparagopsis*, when tested in vitro [4–6], in sheep [7], beef cattle [8, 9], and dairy cows [10, 11]. These studies have reported a wide range of CH_4_ reduction percentages with *Asparagopsis* supplementation. A recent meta-analysis of the effects of feeding predominantly *Asparagopsis* to cattle found a 37% reduction in CH_4_ yield [12], which is similar to the reductions achieved by other methanogenesis inhibitors, such as 3-nitrooxypropanol (22% and 39%, respectively in beef and dairy cattle) [13]. Nevertheless, other recent studies that tested the efficacy of *Asparagopsis* have reported CH_4_ reductions up to 80% [8, 10].

The CH_4_ inhibition by *Asparagopsis* is due to the presence of halogenated methane analogues (HMAs), encapsulated into specialized gland cells until its release as a natural plant defence mechanism [14]. Bromoform (CHBr_3_) has been shown to be the most abundant HMA in *Asparagopsis* as its principal anti-methanogenic compound [15]. The most reported mechanism for the inhibitory activity of HMAs in ruminants is by reacting with reduced vitamin B_12_ of coenzyme M methyltransferase and inhibiting the cobamide-dependent methyl-transferase step of methanogenesis [16, 17]. However, HMAs have also been found to bind to nickel tetrapyrrole (cofactor F430) of methyl-coenzyme M reductase (MCR), thereby inhibiting the reductive release of CH_4_ from methyl-coenzyme M [18–20]. *Asparagopsis taxiformis* is currently considered one of the most promising species due to its high CHBr_3_ content [1 to 15.8 mg/g of biomass on a dry matter (DM) basis] [19] and thus its capability for decreasing CH_4_ production when added at low inclusion rates to forage- and grain-based diets, without negatively affecting daily feed intake, feed conversion efficiencies or rumen function [21]. However, the large variation in the anti-methanogenic effect could be due to the inclusion rate, CHBr_3_ content in the seaweed, the fibre content in the diet received by the animal or the composition of the rumen microbial community [22]. A thorough understanding of how these factors affect the efficacy of *A. taxiformis* in decreasing enteric CH_4_ emissions is essential for the implementation of this mitigation strategy across all the different ruminant production systems.

Bromoform is recognized as an animal carcinogen and has been associated with renal and liver toxicity [23]. However, previous studies have shown no accumulation of CHBr_3_ in animal tissues or milk, taken from beef cattle [8] and dairy cows [10, 11] which had been offered *Asparagopsis* at low inclusion rates. This suggests that CHBr_3_ may be metabolised in the rumen. In contrast, a recent study with no control animals and high inclusion rates of *A. taxiformis* suggested that CHBr_3_ may be excreted in urine and milk in dairy cows [24]. However, its concentration in milk was less than half the United States Environment Protection Agency (U.S. EPA) drinking water standard for CHBr_3_. The transfer of HMAs, including CHBr_3_, into ruminant-derived food products is largely determined by its potential degradation or modification in the rumen environment. Studies have demonstrated that methanogens have the capacity to metabolise HMAs in nature [25–27] because coenzyme M methyltransferase, and specially MCR, can reductively dehalogenate a range of HMAs [28], including CHBr_3_ [17]. However, there is currently no literature on the metabolic fate of brominated halomethanes in the ruminant digestive system and whether the diet received by the animal could have an effect in the HMA degradation process.

Based on experiments that have shown degradation of chlorinated hydrocarbons in vitro in anaerobic sludge [29] and reductive dehalogenation of carbon tetrachloride by cell suspensions of archaea *Methanosarcina barkeri* resulting in chloroform, methylene chloride, methyl chloride, and CH_4_ as intermediates [28], we hypothesised that rumen methanogenic archaea catalyse the dehalogenation of CHBr_3_ to dibromomethane (CH_2_Br_2_), then bromomethane (CH_3_Br), and finally CH_4_ and bromine radical (Br). Therefore, the aim of the present study was to describe the degradation pattern of the active compound CHBr_3_ from *A. taxiformis* using an in vitro rumen simulation model under two dietary conditions representing different rumen fermentation rates. Additionally, the sensitivity of the most relevant methanogen species to CHBr_3_ was evaluated in vitro using seven pure cultures of methanogenic strains.

## Materials and methods

### Exp. 1: In vitro batch culture incubation

Fresh alfalfa hay and barley grain were used as substrate for the incubations. They were ground with a hammer mill (220 v, type WRB 90 Lb/4P, Dietz-motoren KG, Eleckromotorenfabrik, Dettingen unter Teck, Germany) to pass through a 1-mm screen. *Asparagopsis taxiformis* was obtained from SeaExpert (Faial, Portugal) and its CHBr_3_ concentration was 6 mg/g DM. It was freeze-dried and ground using a laboratory mill (IKA All analytical mill, Staufen, Germany) to pass through a 1-mm screen. The mill equipment was previously cooled in liquid nitrogen to avoid overheating and damaging *A. taxiformis* chemical integrity. Afterwards, *A. taxiformis* was ground 2 × 30 s with a 30-s interval between cycles to cool the mill. Milled *A. taxiformis* was stored in an airtight recipient contained in a desiccator and kept at 4 °C. Dry matter (DM), organic matter (OM), crude protein (CP), ether extract (EE), neutral detergent fibre (NDF), acid detergent fibre (ADF) and acid detergent lignin (ADL) of the substrates and the algae were analysed as described in Arco-Pérez et al. [30]. Dry matter (method 934.01) and ash (or OM) (method 942.05) were determined according to AOAC (2005) [31]. The nitrogen values (AOAC method 990.03) were determined using the Dumas method (Leco TruSpec CN, St. Joseph, MI, USA) and converted to CP by multiplying by 6.25. Ether extract was measured by extraction with petroleum ether (AOAC method 920.39). The analyses of NDF and ADF were carried out according to Van Soest et al. [32] using an Ankom 220 fiber analyser unit (Ankom Technology Corp., Macedon, NY, USA), with α-amylase for NDF analysis in concentrate samples, while ADL was determined by solubilisation of cellulose with 72% sulfuric acid. The chemical composition of alfalfa hay, barley grain and *A. taxiformis* is presented in Table 1.

**Table 1:** Chemical composition of the substrates of the diet and *Asparagopsis taxiformis* used in Exp. 1

Nutrients, g/kg DM	Alfalfa hay	Barley grain	*A. taxiformis*
DM	884	905	877
OM	901	975	460
CP	174	134	146
EE	13.7	20.1	2.10
NDF	428	285	194
ADF	303	67.8	66.5
ADL	63.0	8.70	15.3

*DM* Dry matter, *OM* Organic matter, *CP* Crude protein, *EE* Ether extract, *NDF* Neutral detergent fibre, *ADF* Acid detergent fibre, *ADL* Acid detergent lignin

An in vitro batch culture incubation [33] was used to study the degradation of CHBr_3_ naturally present in *A. taxiformis* in the rumen microbial environment. Rumen fluid was collected from five Limousine cows, aged 12 to 14 months, from a commercial slaughterhouse in Granada (Spain). The cows were adapted to a total mixed diet with a 50:50 forage:concentrate ratio in DM basis. The rumen digesta was filtrated through a double layer of cheese cloth and mixed with pre-warmed incubation buffer (0.35 g/L NaHCO_3_, 0.04 g/L (NH_4_)HCO_3_) [34] in a 1:2 ratio. Thereafter, 50 mL of the solution were anaerobically dispensed to 120 mL Wheaton bottles containing 0.5 g DM of the experimental diet.

Two diets differing in forage:concentrate ratio were used as fermentation substrate: 1) one diet with high concentrate proportion (HC; alfalfa hay and barley grain in a 30:70 ratio in DM) to promote a quick fermentation rate; and 2) a second diet high in forage (HF; alfalfa hay and barley grain in a 70:30 ratio in DM) to promote a slower and more sustained microbial fermentation rate. The objective of using two different diets was to assess whether the different fermentation rates could affect the degradation pattern of CHBr_3_. Moreover, for each diet, the inclusion or not of *A. taxiformis* on the top of the diet was considered: 1) *A. taxiformis* supplemented at 0 of substrate on a DM basis (Control, CTL); and 2) *A. taxiformis* supplemented at 2% of substrate on a DM basis (AT). The inclusion rate of *A. taxiformis* at 2% was chosen based on the observed CH_4_ inhibition in a previous in vitro study [35]. The objective of using two different diets was to assess whether the different fermentation rates could affect the degradation pattern of CHBr_3_. A total of 220 Wheaton bottles were used according to the following design: *A. taxiformis* inclusion level (CTL and AT) × substrate (HC and HF) × 11 sampling times for each rumen inoculum (*n* = 5). Different sets of Wheaton bottles were incubated for 0, 0.5, 1, 3, 6, 8, 12, 16, 24, 48 and 72 h to assess the pattern of degradation of CHBr_3_ into secondary metabolites. Immediately after inoculation, bottles were sealed, gently mixed, and kept in an incubator at 39 ºC.

At each sampling time (0, 0.5, 1, 3, 6, 8, 12, 16, 24, 48 and 72 h), headspace gas pressure measurement and headspace gas and culture content collection were performed from each set of Wheaton bottles that corresponded to the designated sampling point. Gas pressure in the headspace was measured using a Wide Range Pressure Meter (Sper Scientific LTD, Scottsdale, AZ, USA) and the entire volume of headspace gas was collected in 250-mL PVDF gas-tight bags (Cole-Parmer Kynar, Vernon Hills, IL, USA) for CH_4_ and H_2_ analyses. To ensure the complete collection of headspace gas, a syringe connected to the pressure transducer was used to extract the gas until the pressure inside the bottle reached zero. The headspace gas from the remaining bottles was released at each point until their sampling time. Samples of 10 mL of liquid culture content were collected at each time point in GC-MS vials to quantify the concentration of halogenated compounds (CHBr_3_ and CH_2_Br_2_). In addition, 0.8 mL liquid samples were collected at the end of the incubation (72 h) and diluted in 0.8 mL of an acid solution (0.5 mol/L HCl, 20 g/L metaphosphoric acid containing 0.8 g/L of crotonic acid as internal standard) to determine VFA concentration and profile. All the samples were stored at −20 ºC immediately after sampling.

The concentrations of CH_4_ and H_2_ in the headspace gas samples were determined using a micro gas chromatography system (Agilent 490, Santa Clara, CA, USA) equipped with two column channels: a 10 m CP-Molsieve 5A column on Ar as carrier gas, and a 10 m CP-PoraPLOT U column on He as carrier gas, following a methodology adapted from Cluett et al. [36]. The system was calibrated using a certified standard gas mix (Messer Gases for Life, Tarragona, Spain) with the following composition: 1% H_2_, 3% O_2_, 20% CH_4_, 26% N_2_, 50% CO_2_. A volume of 10 mL from each sample was directly introduced from the gas bags to the micro GC, using the internal sampling pump. The analysis was performed three times for each individual sample. The GC analyses were carried out at the Instrumental Technical Services of the Estación Experimental del Zaidín (SIC-EEZ), CSIC, Granada, Spain. Gas production was calculated by transforming pressure measurements into volume units using the Ideal Gas Law under standard atmospheric pressure and 39 ºC.

Concentrations of individual VFA (acetate, propionate, isobutyrate, butyrate, isovalerate and valerate) were determined by a GC system coupled with a Flame Ionization Detector (Auto-System PerkinElmer, Norwalk, CT, USA) using a crosslinked 100% polyethylene glycol column (TRB-FFAP, 30 m × 0.53 mm i.d. × 1 µm film thickness, Teknokroma, Spain), as described in Arco-Pérez et al. [30]. One μL of each sample was injected, in split mode (20 mL/min). Nitrogen (1 mL/min) was used as carrier gas, and the injector and detector temperature were 260 °C and 275 °C, respectively. The column temperature was maintained at 100 °C for 1 min, increased at 15 °C/min up to 160 °C, remaining at this point for 1 min. The identification of VFA peaks was based on the retention time of the external standards, using crotonic acid as the internal standard for quantification. Standard curves were prepared by dilution (1:1) of the standard mixture in the same solution in which the rumen content samples were added.

The concentrations of CHBr_3_ and CH_2_Br_2_ were measured as described in Colomb et al. [37]. Samples were analysed by headspace solid-phase micro extraction (CTC Analytics PAL Combi-xt Autosampler, Zwingen, Switzerland) interfaced with two-dimensional gas chromatography and with time-of-flight mass spectrometry (Waters Micromass Quattro micro GC, Milford, MA, USA). Analyte detection took place with mass spectrometry in full scan mode with 500 scans/s. The determination of CHBr_3_ and CH_2_Br_2_ were carried out at the Centre for Scientific Instrumentation of the University of Granada (CIC-UGR), Spain.

### Exp. 2: In vitro pure cultures of methanogenic archaea

The pure cultures of six ruminal methanogens strains (*Methanobrevibacter smithii* DSM 861, *Methanobrevibacter ruminantium* DSM 1093, *Methanosphaera stadtmanae* DSM 3091, *Methanosarcina barkeri* DSM 800, *Methanobrevibacter millerae* DSM 16643 and *Methanobacterium mobile* DSM 1539) and *Methanothermobacter wolfei* DSM 2970 were acquired from DSMZ-German Collection of microorganisms and cell culture. These species were selected to represent some of the most abundant methanogens in the rumen across the main phylogenetic clades [38]. Methanogens cultures were carried out in Hungate tubes with medium and growing conditions as specified by DSMZ for anaerobes. Culture media (119, 120, 161, 322), described in detail at DSMZ website (www.dsmz.de), were prepared anaerobically, aseptically and under an atmosphere consisting of 80% H_2_ and 20% CO_2_ in anaerobic chamber (Whitley DG250 Anaerobic Workstation, Don Whitley Scientific Limited, West Yorkshire, UK). For the inoculation, ampoules with the different pure cultures were handled within an anaerobic chamber and under an atmosphere consisting of 80% H_2_ and 20% CO_2_ as specified by DSMZ. Two Hungate tubes containing 5 mL of the corresponding specific medium of every strain were used for the following treatments: control (CTL; no treatment applied), 100 µmol/L 2-bromoethanesulphonate (BES), as positive control of inhibition used in previous works [39], and increasing concentrations of CHBr_3_ (0.4, 2, 10 and 50 µmol/L). The dose range was determined to include the inclusion rate of *A. taxiformis* used in Exp. 1 (2% DM diet which is equivalent to 40 µmol/L). The compounds BES and CHBr_3_ were obtained from Sigma-Aldrich (Merck KGaA, Darmstadt, Germany). Stock solutions of BES (100 µmol/L) and CHBr_3_ (50 µmol/L) were prepared and stored at 4 ºC.

Cultures were incubated for 14 d, and the corresponding treatment was applied on d 2. Three consecutive incubation batches were run (*n* = 3) with analytical duplicates for each treatment that were averaged. Pressurization with H_2_/CO_2_ gas in anaerobic chamber was applied to achieve 1 bar in the tubes’ headspace. Tubes were horizontally placed in a shaking incubator at 37 ºC at 120 r/min in the dark. Methanogen growth was followed by CH_4_ production [40]. On d 2, 4, 6, 8, 10, 12 and 14 of incubation, 0.5-mL samples of gas produced were taken and immediately injected in a flame ionization-detection GC (HP Hewlett 5890, Packard Series II, Waldbronn, Germany) using a 0.5-mL Sample-Lock syringe (Hamilton, Nevada, USA) for CH_4_ analysis [41]. The concentration of CH_4_ was determined using a standard curve generated by injecting different volumes of 99.9% pure CH_4_ pre and post the injection of samples. After that, H_2_/CO_2_ gas was added to each tube to maintain 1 bar pressure.

### Statistical analyses

Before conducting the ANOVA, the assumptions of the normality and homogeneity of the variance for Exp. 1 and 2 were checked using the Shapiro–Wilk and the Bartlett’s tests, respectively. Results from in vitro batch cultures (Exp. 1) were statistically analysed by a 2 × 2 factorial ANOVA:

$${Y}_{ijk} = \mu + {R}_{i} + {D}_{j} + {(R \times D)}_{ij} + {A}_{k} + {e}_{ijk}\, (\mathrm{one\, for\, each\, time\, point})$$

Where *Y*_*ijk*_ represents the dependent, continuous variable, *µ* is the overall population mean, *R*_*i*_ is the fixed effect of the *A. taxiformis* inclusion rate (CTL vs. AT), *D*_*j*_ is the fixed effect of the diet (HC vs. HF), (R × D)_*ij*_ represents the interaction term, *A*_*k*_ represents the random effect of the animal inocula (*n* = 5), and *e*_*ijk*_ is the residual error.

Results from methanogenic archaea pure cultures (Exp. 2) were statistically analysed by repeated measures as follows:

$${Y}_{ijk} = \mu + {C}_{i} + {T}_{j} + {(C \times T)}_{ij} + {A}_{k} + {e}_{ijk}\, (\mathrm{one\, for\, each\, archaea\, pure\, culture})$$

Where *Y*_*ijk*_ represents the dependent, continuous variable, *µ* is the overall population mean, *C*_*i*_ is the fixed effect of CHBr_3_ concentration (0, 0.4, 2, 10, 50 µmol/L), *T*_*j*_ is the fixed effect of the time (2, 4, 6, 8, 10, 12 and 14 d), (C × T)_*ij*_ represents the interaction term, *A*_*k*_ represents the repeated run (*n* = 3), and *e*_*ijk*_ is the residual error.

When significant effects were detected, polynomial contrasts were used to determine linear (L) and/or quadratic (Q) responses in Exp. 2, and means were compared by Fisher’s protected LSD test when significant interactions were found (*P* < 0.05), using the StatGraphics Centurion 19 software (StatPoint Technologies, Inc. USA, 2020). Significant effects were declared at *P* < 0.05 and tendencies to differences at *P* < 0.10.

## Results

### Exp. 1: In vitro batch culture incubation

#### Fermentation profile

The results from the in vitro gas production test confirmed that different fermentation pattern was obtained by using either HC or HF diets (Table 2). This was reflected not only in significantly greater volume of gas produced by HC diet (*P* = 0.046) but also a significantly greater gas production rate (*P* = 0.046) at most time intervals. No significant differences were noted in total VFA production (*P* = 0.243), but the HC diet promoted a higher molar proportion of propionate (*P* = 0.001), butyrate (*P* = 0.008) and valerate (*P* = 0.021), whereas the HF diet promoted a higher acetate molar proportion (*P* < 0.001) (Table 3).

**Table 2:** Effect of the inclusion rate (R) of *Asparagopsis taxiformis* and the type of diet (D) on the in vitro cumulative gas production and production rate at different incubation intervals (Exp. 1)

	HC		HF		SEM	*P*-value		
	CTL	AT	CTL	AT		R	D	R × D
Cumulative gas production, mL								
0.5 h	6.10	4.60	5.60	4.30	0.18	< 0.001	0.046	0.560
1.5 h	15.2	10.9	14.5	10.0	0.40	< 0.001	0.092	0.812
3 h	25.7	19.0	24.3	18.0	0.60	< 0.001	0.079	0.731
6 h	41.5	31.6	37.3	29.0	1.21	< 0.001	0.02	0.505
8 h	52.3	40.8	44.6	36.0	1.65	< 0.001	0.004	0.395
12 h	66.4	52.5	55.2	44.3	1.37	< 0.001	< 0.001	0.309
16 h	83.6	66.9	69.1	55.8	2.19	< 0.001	< 0.001	0.461
24 h	93.3	75.6	77.5	63.2	2.36	< 0.001	< 0.001	0.488
48 h	115	94.0	105	85.9	2.74	< 0.001	0.007	0.654
72 h	130	106	123	102	3.16	< 0.001	0.108	0.772
Gas production rate, mL/h								
0–0.5 h	12.23	9.27	11.18	8.65	0.362	< 0.001	0.046	0.560
0.5–1.5 h	9.09	6.23	8.96	5.69	0.300	< 0.001	0.297	0.508
1.5–3 h	6.98	5.39	6.48	5.30	0.312	0.002	0.367	0.523
3–6 h	5.29	4.20	4.34	3.67	0.227	0.004	0.010	0.380
6–8 h	5.37	4.62	3.64	3.51	0.281	0.153	< 0.001	0.293
8–12 h	3.52	2.93	2.66	2.07	0.139	0.002	< 0.001	0.982
12–16 h	4.31	3.59	3.49	2.87	0.260	0.030	0.016	0.851
16–24 h	1.21	1.08	1.05	0.93	0.066	0.088	0.044	0.969
24–48 h	0.91	0.77	1.13	0.94	0.032	< 0.001	< 0.001	0.579
48–72 h	0.62	0.51	0.79	0.66	0.042	0.023	0.005	0.753

*HC* High-concentrate diet, *HF* High-forage diet, *CTL* Control (*Asparagopsis taxiformis* at 0 DM), *AT Asparagopsis taxiformis* at 2% DM, *SEM* Standard error of the mean

**Table 3:** Effect of the inclusion rate (R) of *Asparagopsis taxiformis* and the type of diet (D) on rumen fermentation parameters at 72 h of incubation (Exp. 1)

	HC		HF		SEM	*P*-value		
	CTL	AT	CTL	AT		R	D	R × D
Total VFA, mmol/L	82.2	80.1	78.0	72.8	4.580	0.446	0.243	0.745
VFA, mol/100 mol								
Acetate	62.9	52.4	66.7	56.0	0.347	< 0.001	< 0.001	0.886
Propionate	21.3	28.6	19.2	27.5	0.366	< 0.001	0.001	0.218
Butyrate	9.07	12.13	8.35	10.7	0.310	< 0.001	0.008	0.314
Isobutyrate	1.75	1.83	1.57	1.40	0.100	0.631	0.015	0.241
Isovalerate	2.85	2.38	2.45	2.05	0.208	0.065	0.115	0.866
Valerate	2.05	2.63	1.78	2.32	0.103	< 0.001	0.021	0.898
A:P	2.96^b^	1.84^c^	3.49^a^	2.05^c^	0.038	< 0.001	< 0.001	0.003

*HC* High-concentrate diet, *HF* High-forage diet, *CTL* Control (*Asparagopsis taxiformis* at 0 DM), *AT Asparagopsis taxiformis* at 2% DM, *SEM* Standard error of the mean, *VFA* Volatile fatty acids, *A:P* Acetate:propionate ratio

^a−c^Values within a row with different superscripts differ significantly at *P* < 0.05 due to R × D interaction

The addition of AT significantly decreased the cumulative gas production (Table 2) at every time interval (*P* < 0.001), but not the VFA production (Table 3) after 72 h incubation (*P* = 0.446). Additionally, AT treatment had a strong effect on fermentation pattern promoting an increase in the molar proportions of propionate, butyrate and valerate (*P* < 0.001), and a decrease in the molar proportion of acetate (*P* < 0.001).

Table 3 also shows a significant interaction (*P* = 0.003) between *A. taxiformis* inclusion rate and the type of diet (R × D) for the acetate:propionate ratio (A:P) indicating a larger decrease for the HF than for the HC diet.

#### Methane and dihydrogen production

The main effect of AT inclusion on rumen fermentation was reflected in a substantial inhibition of CH_4_ cumulative production (*P* = 0.002) and production rate (*P* = 0.018) for both diets from 1.5 to 72 h of incubation (Table 4). During the initial incubation period (0–1.5 h), a significant interaction (*P* = 0.029) showed that the inhibition of CH_4_ cumulative production and production rate were only detected with the HF diet.

**Table 4:** Effect of the inclusion rate (R) of *Asparagopsis taxiformis* and the type of diet (D) on the in vitro CH_4_ cumulative production and production rate at different incubation intervals (Exp. 1)

	HC		HF		SEM	*P*-value		
	CTL	AT	CTL	AT		R	D	R × D
Cumulative CH_4_, mL								
1.5 h	0.08^ab^	0.05^bc^	0.13^a^	0.00^c^	0.017	0.001	0.93	0.029
6 h	1.32	0.08	0.77	0.02	0.185	< 0.001	0.137	0.218
12 h	1.95	0.12	1.85	0.03	0.329	< 0.001	0.771	0.984
24 h	3.18	0.20	4.14	0.04	0.833	0.002	0.644	0.517
72 h	8.36	0.52	13.2	0.15	1.833	< 0.001	0.252	0.187
CH_4_ production rate, µL/h								
0–1.5 h	56.3^ab^	33.3^bc^	85.2^a^	2.30^c^	11.50	0.001	0.93	0.029
1.5–6 h	274	5.91	143	3.62	38.70	< 0.001	0.119	0.130
6–12 h	106	7.71	180	1.88	36.60	0.004	0.379	0.307
12–24 h	102	6.38	191	0.41	49.70	0.018	0.427	0.366
24–72 h	108	6.78	189	2.40	30.80	0.001	0.244	0.199

*HC* High-concentrate diet, *HF* High-forage diet, *CTL* Control (*Asparagopsis taxiformis* at 0 DM), *AT Asparagopsis taxiformis* at 2% DM, *SEM* Standard error of the mean

^a−c^Values within a row with different superscripts differ significantly at *P* < 0.05 due to R × D interaction

This strong inhibition of methanogenesis resulted in increased H_2_ production rate, which was significantly higher (*P* = 0.006) in AT than in CTL from 1.5 to 12 h of incubation (Table 5). Hydrogen cumulative production was significantly increased (*P* = 0.020) for HC compared to HF diet at 12 h of incubation. From 24 h until the end of the incubation period, the effect of AT on H_2_ cumulative production significantly differed (significant interaction, *P* < 0.032) based on the type of diet, with a greater increase observed when AT was included with HC diet.

**Table 5:** Effect of the inclusion rate (R) of *Asparagopsis taxiformis* and the type of diet (D) on the in vitro H_2_ cumulative production and production rate at different incubation intervals (Exp. 1)

	HC		HF		SEM	*P*-value		
	CTL	AT	CTL	AT		R	D	R × D
Cumulative H_2_, µL								
1.5 h	3.91	0.23	0.10	0.06	1.937	0.361	0.331	0.371
6 h	4.69	5.08	0.30	2.88	2.190	0.514	0.166	0.629
12 h	5.20	23.3	0.52	8.66	3.410	0.004	0.020	0.178
24 h	8.38^c^	107^a^	1.64^d^	26.7^b^	14.56	0.002	0.015	0.032
72 h	8.83^c^	108^a^	2.11^d^	27.9^b^	14.34	0.002	0.014	0.031
H_2_ production rate, µL/h								
0–1.5 h	2.61	0.15	0.07	0.04	1.291	0.361	0.331	0.371
1.5–6 h	0.17	1.08	0.04	0.63	0.205	0.006	0.191	0.450
6–12 h	0.08	3.03	0.04	0.96	0.540	0.006	0.082	0.094
12–24 h	0.27^c^	6.99^a^	0.09^d^	1.51^b^	1.011	0.003	0.021	0.028
24–72 h	0.01	0.02	0.01	0.02	0.010	0.274	0.698	0.726

*HC* High-concentrate diet, *HF* High-forage diet, *CTL* Control (*Asparagopsis taxiformis* at 0 DM), *AT Asparagopsis taxiformis* at 2% DM, *SEM* Standard error of the mean

^a−d^Values within a row with different superscripts differ significantly at *P* < 0.05 due to R × D interaction

#### Bromoform degradation pattern

**Fig. 1:**
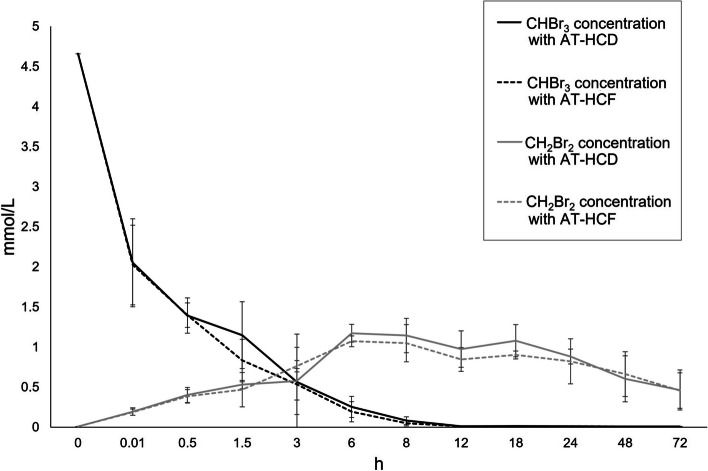
Effect of supplementing with *Asparagopsis taxiformis* at 2% DM (AT) to high-concentrate (HC) and high-forage (HF) diets on the in vitro concentration (mmol/L) of bromoform (CHBr_3_) and dibromomethane (CH_2_Br_2_) at different incubation times (h)

The monitorization of CHBr_3_ and CH_2_Br_2_ concentrations throughout the incubation process is showed in Fig. 1. A rapid degradation of CHBr_3_ was observed, with 70% degraded within the first 30 min and nearly 90% after 3 h of incubation. Most (> 99%) of the CHBr_3_ was degraded within 12 h of incubation.

In line with the observations on CHBr_3_ degradation, CH_2_Br_2_ was not detected at 0 h but its presence was quickly detected in the samples after the incubation began and increasing rapidly until it peaked at 6–8 h of incubation. Then, a gradual decline in concentration was observed towards the end of the incubation period (18 to 72 h) at which around 50% of the maximum CH_2_Br_2_ detected at 6 h was no longer present in the samples. Neither CHBr_3_ degradation nor CH_2_Br_2_ synthesis were significantly affected by the type of diet used as substrate (HC or HF).

### Exp. 2: In vitro pure cultures of methanogenic archaea

**Fig. 2:**
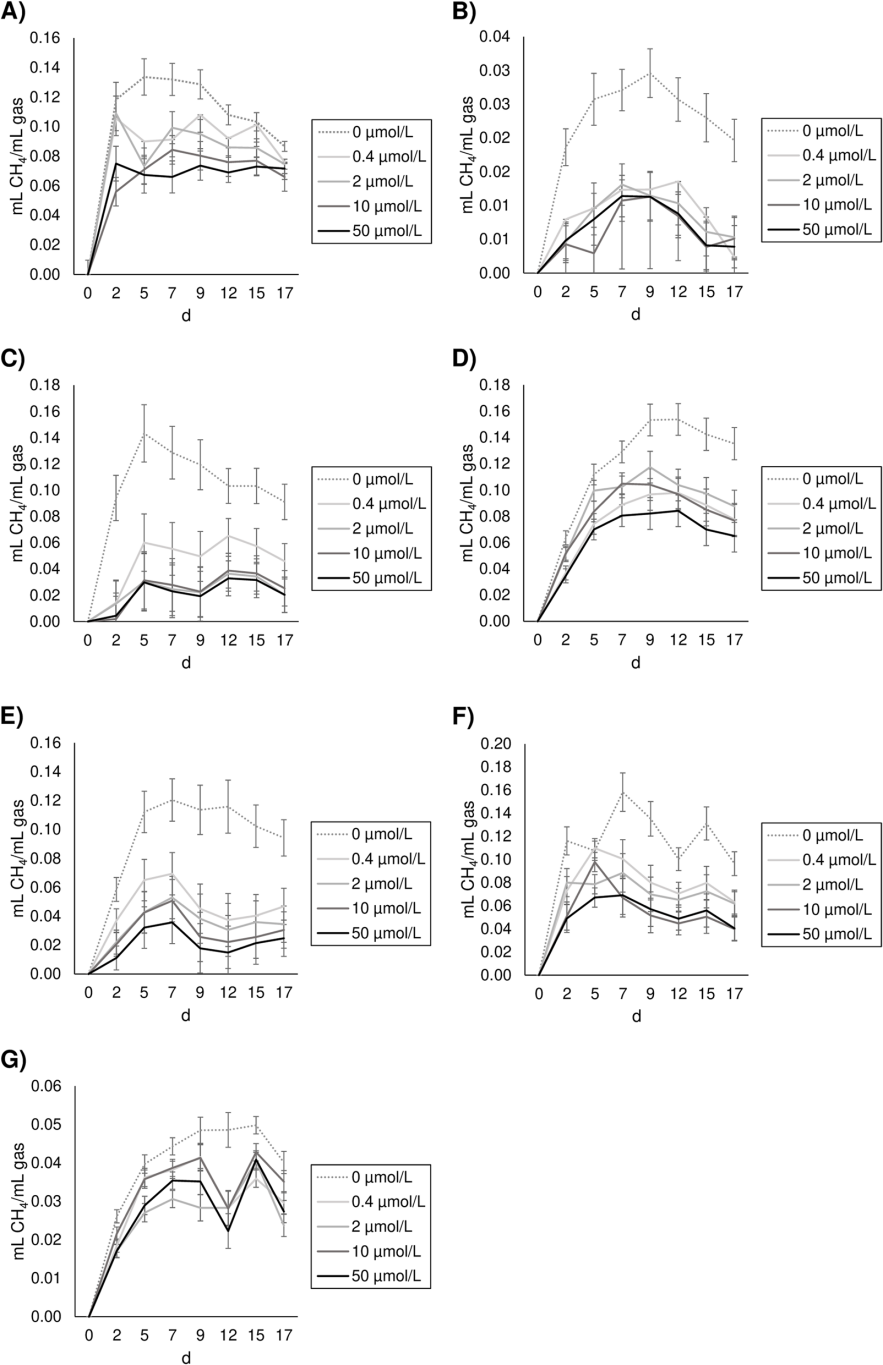
Effect of bromoform (CH_3_Br) at different concentrations (0.4, 2, 10 and 50 µmol/L) on the growth (mL CH_4_/mL gas) of *Methanobrevibacter smithii* (**A**), *Methanobrevibacter ruminantium* (**B**), *Methanosphaera stadtmanae* (**C**), *Methanosarcina barkeri* (**D**), *Methanobrevibacter millerae* (**E**) *Methanothermobacter wolfei* (**F**) and *Methanobacterium mobile* (**G**)

The increasing addition of CHBr_3_ promoted a quadratic response in the inhibition of the growth of *M. smithii*, *M. ruminantium*, *M. stadtmanae*, *M. millerae*, *M. wolfei*, and *M. mobile,* with the highest drop observed for concentrations between 2 and 10 µmol/L, depending on the methanogens species. Moreover, a linear dose-response was observed for *M. barkeri* (Table 6; Fig. 2). *Methanosphaera stadtmanae* (Fig. 2C) and *M. ruminantium* (Fig. 2C) were the most affected strains to the highest concentrations of CHBr_3_ (50 µmol/L), while *M. smithii* (Fig. 2A) and *M. mobile* (Fig. 2G) could be considered the most resistant species to the anti-methanogenic activity of CHBr_3_, as their growing rates were only decreased by around 30%. The incubation time also modulated the CH_4_ inhibition promoting a linear decrease for *M. smithii* and linear increases for *M. ruminantium* and *M. barkeri,* while quadratic responses were noted for *M. stadtmanae* and *M. millerae*.

**Table 6:** Effect of bromoform at different concentrations (C) and incubation times (T) on CH_4_ inhibition rate (%) in different archaeal species (Exp. 2)

	BES 100 µmol/L	CHBr_3_ concentration, µmol/L				SEM	Contrasts C	Time							SEM	Contrasts T
		0.4	2	10	50			d 2	d 5	d 7	d 9	d 12	d 15	d 17		
*Methanobrevibacter smithii*	17.8 ± 3.46	14.3^b^	19.0^b^	34.4^a^	32.9^a^	2.96	L^**^, Q^***^	27.5^abc^	35.4^a^	32.3^ab^	28.5^abc^	20.0^bcd^	17.7^cd^	14.5^d^	3.92	L^**^
*Methanobrevibacter ruminantium*	60.3 ± 4.05	58.4	62.9	71.1	68.7	3.98	Q^†^	54.2^b^	72.2^ab^	54.5^b^	60.3^bc^	60.3^bc^	76.1^a^	79.1^a^	5.26	L^**^
*Methanosphaera stadtmanae*	31.2 ± 3.35	54.8^b^	73.7^a^	75.4^a^	76.6^a^	1.71	L^*^, Q^**^	89.9^a^	72.7^b^	73.8^b^	74.0^b^	56.7^c^	58.2^c^	65.7^bc^	2.26	L^***^, Q*
*Methanosarcina barkeri*	40.3 ± 3.81	37.6^ab^	24.7^b^	31.8^ab^	46.5^a^	3.28	L^*^	29.9	28.7	28.5	35.0	38.9	40.8	44.2	4.34	L^†^
*Methanobrevibacter millerae*	28.7 ± 5.14	42.0^b^	63.3^a^	69.2^a^	75.6^a^	5.56	L^**^, Q^*^	47.5^c^	54.6^bc^	53.4^bc^	71.6^ab^	77.1^a^	69.9^ab^	63.8^abc^	7.35	L^*^, Q^†^
*Methanothermobacter wolfei*	19.6 ± 5.75	30.5^b^	39.9^ab^	48.6^a^	48.4^a^	3.42	L^*^, Q^**^	43.3^a^	20.5^b^	47.9^a^	50.6^a^	40.0^a^	42.7^a^	47.8^a^	4.52	NS
*Methanobacterium mobile*	10.2 ± 4.05	22.3^b^	33.3^a^	19.9^b^	30.1^a^	2.67	Q^†^	30.7^b^	20.1^d^	16.0^d^	23.5^bcd^	43.7^a^	20.6^cd^	30.3^bc^	3.53	NS

In the first column, mean ± SE (standard error). For the treatments, SEM (standard error of the mean)

BES treatment was not included in the statistical analysis

*BES* 2-bromoethanesulphonate, *CHBr*_*3*_ Bromoform, *SEM* Standard error of the mean

^a−d^Values within a row with different superscripts differ significantly at *P* < 0.05 (*n* = 3)

Contrast: *NS* Not significant, *L* Linear response, *Q* Quadratic response; ^***^P < 0.001; ^**^P < 0.01, ^*^P < 0.05; ^†^*P* < 0.1

## Discussion

### Microbial fermentation profile

The type of diet consumed by the ruminant, and particularly the forage:concentrate ratio, determines to a great extent the fermentation pattern in the rumen [42]. There is a faster fermentation rate of carbohydrates when the proportion of concentrate in the diet is increased, whereas diets mainly based on forage have a high content in structural carbohydrates such as cellulose and hemicellulose, which require longer times to be degraded by rumen microorganisms. Our study showed that HC diet resulted in a greater volume of gas produced compared with HF diet, suggesting a greater yield of fermentation products when concentrate proportion is increased [43]. The differences between diets were reflected in a shift in A:P and the fermentation rate. A greater supply of fermentable carbohydrates leads to a faster fermentation rate, increasing microbial growth and thus enhancing the degradation potential of different substrates. Hagemeister et al. [44] showed an increase of 22.1 g of microbial protein biomass per 100 g increase in fermentable organic matter supplied to the rumen.

According to Roque et al. [45], the inclusion of AT had an effect on in vitro cumulative gas production. In our study, the efficacy of AT to decrease the volume of gas produced was detected at the beginning of the fermentation and remained consistent during the 72 h incubation period. Our results differ with those from Machado et al. [46] since AT did not decrease total VFA production. This discrepancy could be explained by the far greater level of inclusion used by Machado et al. [46], which reached up to 16.7% of total OM in the diet. However, both studies agree with the finding that AT changed the VFA profile. Acetate molar proportion decreased whereas that of propionate and butyrate increased, decreasing A:P as reported by Roque et al. [45], Machado et al. [46] and Kinley et al. [47]. When methanogenesis is inhibited, an increase in propionate production is normally observed because pyruvate is reduced to propionate in one of two multi-step pathways [48].

### Methanogenesis

Consistent with previous works [7–9, 11], the present study demonstrated the anti-methanogenic activity of *A. taxiformis*. The 97% reduction of CH_4_ production was similar to the level of reduction observed in a previous in vitro study with the same batch of *A. taxiformis* and inclusion rate [35]. Machado et al. [46] reported 85% reduction of CH_4_ production with 1% (OM basis) inclusion rate in the diet and a nearly total reduction at doses above 2%. Kinley et al. [47] observed that no detectable CH_4_ was produced with 2% (OM basis) *A. taxiformis* inclusion after 24 h. Roque et al. [45] reported a 95% reduction in CH_4_ production when the dosage was increased to 5% OM. It is noticeable that the literature reflects that the methanogenesis inhibition rate achieved by the addition of AT is rather variable, which could potentially be due to different factors such as differences in AT inclusion rate, animal species, diet formulation and CHBr_3_ content of the macroalgae, which in turn depends on its geographical origin, harvesting season and storage conditions [22].

As described above, the lower forage:concentrate ratio in the diet shifted the fermentation profile from acetate towards propionate production. As the proportion of forage in the diet increases, the proportion of acetate rises and propionate decreases, which means a displacement from H_2_-incorporating propionate to H_2_-producing acetate production [49]. Whereas higher dietary content of fibre increases H_2_ formation, promoting the methanogenic activity of rumen archaea, diets with higher content of starch deprive methanogens of H_2_ and thus decrease CH_4_ production [50].

### Dihydrogen formation

The substantial reduction of CH_4_ production by the addition of AT was accompanied by an increase in rumen H_2_ accumulation, in agreement with previous studies that used AT [8, 9] and other anti-methanogenic feed additives [51, 52]. Elevation of H_2_ was expected as methanogenesis is the main metabolic H_2_ sink in the rumen. When CH_4_ production is inhibited, rumen H_2_ partial pressure increases and, above a certain threshold, rumen fermentation can be potentially compromised [53]. There are regulatory mechanisms to avoid rumen excess H_2_, which involve redirecting part of it towards alternative metabolic pathways such as H_2_-incorporating propionate production [54]. This could explain the alteration of A:P previously observed in this study. However, the propionate production pathway was not efficient enough to capture all the available H_2_ that otherwise would be utilised for the production of CH_4_, and most of the excess H_2_ was then released into the gas phase. This observation suggests that there is still room for developing strategies to capture this H_2_ into valuable fermentation products for the ruminant.

Interestingly, the H_2_ production was also influenced by the type of diet after 12 h of fermentation. Greater amounts of H_2_ were induced by HC diet compared to HF diet. This agrees with Vyas et al. [52] that showed greater emissions of H_2_ by methanogenesis-inhibited animals fed HC diet compared with a mixed forage:concentrate diet. According to Martinez-Fernandez et al. [55], the rumen microbiota utilised more H_2_ available from methanogenesis inhibition when the proportion of forage in the diet was increased. This is possibly due to the slower fermentation rate of the HF diet which might allow a more efficient utilisation of the H_2_ released, compared with the highly fermentable HC diet which might produce H_2_ faster than the rumen methanogens are able to consume.

### Bromoform degradation

Bromoform is the most abundant halogenated metabolite in *A. taxiformis* that provides anti-methanogenic activity to the macroalgae [15]. For *A. taxiformis* to be considered a practical tool for CH_4_ production mitigation, it is essential to guarantee that food products from livestock fed the macroalgae are safe for consumption and that elevated CHBr_3_ levels are not detected in animal tissues or products. No CHBr_3_ residues have been found in samples of kidney, liver, faeces, fat, muscle tissue or milk taken from sheep [7] and beef cattle [8, 9] fed diets with AT at inclusion levels from 0.2% to 1% DM. This suggests that CHBr_3_ is either degraded in the digestive tract or, if absorbed into the animal tissues, is converted to other metabolites.

As reviewed by Glasson et al. [19], studies with methanogens have demonstrated that coenzyme M methyltransferase and MCR reductively dehalogenate a range of HMAs to CH_4_ and other less halogenated intermediates, with cofactor F430 of MCR 50 times more active than coenzyme M methyltransferase [28]. The efficiency of HMA dehalogenation also increases according to expected carbon-halogen bond dissociation energies which decrease in the order F > Cl > Br > I. Therefore, CHBr_3_ would be more efficiently dehalogenated than chloroform. Based on Van Eekert et al. [29] that described chlorinated degradation in vitro in anaerobic sludge, we hypothesized that CHBr_3_ was degraded to CH_2_Br_2_, then CH_3_Br, and finally CH_4_ and Br. Our work demonstrated the dehalogenation process of CHBr_3_ to CH_2_Br_2_ by rumen microbes. We observed that more than 3 mmol/L CHBr_3_ were degraded within 30 min, whereas CH_2_Br_2_ only reached a concentration of 0.4 mmol/L by 30 min and never reached a concentration higher than 1.2 mmol/L in both diets. This suggests that either CHBr_3_ is being degraded through a non-CH_2_Br_2_ pathway or that CH_2_Br_2_ generated over the first 30 min had an extremely short half-life and the experimental approach used was not able to detect its production. The latter conclusion would be consistent with a degradation mechanism that significantly depleted cofactor F_430_ within the first 30 min of fermentation, resulting in a long-term inhibition of CH_4_ production and a long residence time for HMA in the latter hours of the experiment. Cofactor F_430_ is an essential prosthetic group of MCR, which catalyses the last step of methanogenesis [27]. In the actual rumen environment, some amount of F_430_ regeneration would be expected, as would complete degradation of the HMAs. The lack of detection of CH_3_Br and Br as intermediate and end-product of the degradation process, respectively, might be explained by the high volatility of CH_3_Br and the high reactivity of Br with different compounds under the rumen fermentation conditions and the difficulty to use a samples collection procedure from the fermentation vessels to ensure their detection. This deserves further analytical evaluation in future studies.

Our results showed that neither CHBr_3_ degradation nor CH_2_Br_2_ synthesis were significantly affected by the type of diet used as substrate, suggesting that the overall rumen fermentation rate is not a driving factor involved in CHBr_3_ degradation, therefore, similar conclusions can be achieved regardless the diet used by the animal.

Further in vivo investigations are needed to validate the suggested pathway for CHBr_3_ degradation in the rumen. The inclusion rate of *A. taxiformis* should be adjusted to in vivo conditions, as it should not exceed 1% DM due to reductions in feed intake at higher inclusion levels [10, 11]. Considering the rapid degradation of CHBr_3_ observed in vitro and the longer residence time of the digesta in the rumen [56], it is likely that the same results would be observed in vivo. If this is confirmed in vivo, it would provide evidence that CHBr_3_ undergoes rapid degradation upon administration in the rumen, and therefore would not be transferred to animal tissues, milk or urine.

### Methanogenic archaea

Rumen methanogens are microbes capable of producing energy through the reduction of CO_2_ to CH_4_ with consumption of H_2_ from anaerobic fermentation [1]. Mitigation strategies based on the use of HMAs, such as CHBr_3_, whose mechanism of action consists of directly blocking one or more steps of the methanogenic process, are depriving methanogens of their main source of energy [54]. Therefore, inhibition of methanogenesis potentially leads to a reduction in the growth of methanogenic archaeal population [57]. In vitro trials reported significant decreases of the abundance of methanogens with the inclusion of *A. taxiformis* [45, 58] or others synthetic HMA inhibitors [55, 59]. However, the rumen methanogens population is diverse and variable [60] and not all species might be equally sensitive to CHBr_3_ as the key active compound of *A. taxiformis*.

Pure cultures of seven strains of methanogenic archaea, six representing some of the most abundant in the rumen, were used in the present study to evaluate the impact of increasing doses of CHBr_3_ on their individual growth. The minimum concentration tested (0.4 µmol/L CHBr_3_) was sufficient to decrease the growth of all species but not at the same reduction level. For instance, the growth of *M. ruminantium* and *M. stadtmanae* were reduced by more than 50% at that dosage. Previous studies [39, 61] reported the high sensitivity of *M. ruminantium* to BES, another specific inhibitor of the last step of the methanogenesis pathway. However, no significant decrease in *M. ruminantium* growth was observed when CHBr_3_ concentration was further increased. Conversely, *M. smithii, M. stadtmanae, M. barkeri, M. millerae, M. wolfei* and *M. mobile* growing rates were affected in a dose-dependent manner, reaching different rates of CH_4_ inhibition as level of inclusion of CHBr_3_ increased. Both *M. mobile* and *M. smithii* could be considered the most resistant strains to CHBr_3_ as their growing rate was less affected than the others. Similar differences among methanogenic archaea species in response to 3-nitrooxypropanol supplementation were observed by Duin et al. [62].

Differences in the sensitiveness of methanogenic archaea to methanogenesis inhibitors have been attributed to some extent to the varying ability to uptake these inhibitors into the cells [63]. The genus *Methanobrevibacter*, which is the most dominant member of the rumen archaeal community, can be divided into two subgroups according to their expression of MCR that catalyses the rate limiting step of methanogenesis [64]. *Methanobrevibacter smithii* belongs to the clade capable of synthesising both forms of MCR (MCRI and MCRII) while *M. ruminantium* only possesses MCRI [65]. Therefore, *M. ruminantium* methanogenesis pathway is more limited and can be more rapidly blocked by HMA inhibitors, while *M. smithii* has greater MCR activity and requires a higher concentration of inhibitors such as CHBr_3_ to be strongly affected. For the rest of methanogens studied here, it could be suggested that their sensitivity may depend on the saturation capacity of their MCR enzymatic system. Further research on MCR specific characteristics of other species could help to fully understand the impact that CHBr_3_ has on the rumen archaeal population.

Furthermore, the composition of the rumen methanogens community is largely influenced by the basal diet, ruminant species, management, and geographical area [60]. Therefore, it could be expected that the variable relative abundance of the different species of methanogens in the rumen ecosystem, as a consequence of the above listed factors, determined the inhibitory potential of CHBr_3_ containing additives. Consequently, variations in all of these factors could indirectly affect the CH_4_ inhibition rate reached by mitigation strategies based on *A. taxiformis* supplementation. A complete understanding of the rumen microbiome in response to *A. taxiformis* supplementation in animals under different dietary conditions may allow accurate predictions of treatment efficacy.

## Conclusions

Our results show that under in vitro conditions CHBr_3_ is quickly degraded in the rumen, with 90% broken down within the first 3 h. The diet used as substrate does affect neither CHBr_3_ degradation nor CH_2_Br_2_ synthesis, suggesting the fermentation rate is not a driving factor involved in CHBr_3_ degradation. Using pure cultures, CHBr_3_ is shown to inhibit the growth of methanogenic archaea at very low concentrations with some differences among species, which deserve more research to fully understand the potential effectiveness across different dietary regimes. These findings can help to uncover the mode of action of the dietary supplementation with *A. taxiformis* as one of the most promising CH_4_ mitigation strategies in ruminant production.

## Data Availability

All data generated and/or analysed during the current study are available from the corresponding author on reasonable request.

## Abbreviations

ADF: Acid detergent fibre
ADL: Acid detergent lignin
A:P: Acetate:propionate ratio
AT: *Asparagopsis taxiformis* at 2% DM
BES: 2-Bromoethanesulphonate
Br: Bromine
CHBr_3_: Bromoform
CH_2_Br_2_: Dibromomethane
CH_3_Br: Bromomethane
CH_4_: Methane
CO_2_: Carbon dioxide
CP: Crude protein
CTL: Control (*Asparagopsis taxiformis* at 0 DM)
DM: Dry matter
EE: Ether extract
H_2_: Dihydrogen
HC: High-concentrate diet
HF: High-forage diet
HMAs: Halogenated methane analogues
MCR: Methyl-coenzyme M reductase
NDF: Neutral detergent fibre
OM: Organic matter
SEM: Standard error of the mean
VFA: Volatile fatty acids
